# Acute, reproductive, and developmental toxicity of essential oils assessed with alternative in vitro and in vivo systems

**DOI:** 10.1007/s00204-020-02945-6

**Published:** 2020-11-07

**Authors:** Peter Lanzerstorfer, Georg Sandner, Johannes Pitsch, Bianca Mascher, Tobias Aumiller, Julian Weghuber

**Affiliations:** 1 Center of Excellence Food Technology and Nutrition, University of Applied Sciences Upper Austria, Stelzhamerstraße 23, 4600 Wels, Austria; 2 Austrian Competence Centre for Feed and Food Quality, Safety and Innovation, Head Office: FFoQSI GmbH, Technopark 1C, 3430 Tulln, Austria; 3 Delacon Biotechnik GmbH, Langwiesen 24, 4209 Engerwitzdorf, Austria

**Keywords:** Toxicological assessment, In vivo, *C. elegans*, HET-CAM, Essential oil

## Abstract

Essential oils (EOs) have attracted increased interest for different applications such as food preservatives, feed additives and ingredients in cosmetics. Due to their reported variable composition of components, they might be acutely toxic to humans and animals in small amounts. Despite the necessity, rigorous toxicity testing in terms of safety evaluation has not been reported so far, especially using alternatives to animal models. Here, we provide a strategy by use of alternative in vitro (cell cultures) and in vivo (*Caenorhabditis elegans*, hen’s egg test) approaches for detailed investigation of the impact of commonly used rosemary, citrus and eucalyptus essential oil on acute, developmental and reproductive toxicity as well as on mucous membrane irritation. In general, all EOs under study exhibited a comparable impact on measured parameters, with a slightly increased toxic potential of rosemary oil. In vitro cell culture results indicated a concentration-dependent decrease of cell viability for all EOs, with mean IC_50_ values ranging from 0.08 to 0.17% [v/v]. Similar results were obtained for the *C. elegans* model when using a sensitized *bus-5* mutant strain, with a mean LC_50_ value of 0.42% [v/v]. In wild-type nematodes, approximately tenfold higher LC_50_ values were detected. *C. elegans* development and reproduction was already significantly inhibited at concentrations of 0.5% (wild-type) and 0.1% *(bus-5)* [v/v] of EO, respectively. Gene expression analysis revealed a significant upregulation of xenobiotic and oxidative stress genes such as *cyp-14a3*, *gst-4*, *gpx-6* and *sod-3*. Furthermore, all three EOs under study showed an increased short-time mucous membrane irritation potential, already at 0.5% [v/v] of EO. Finally, GC–MS analysis was performed to quantitate the relative concentration of the most prominent EO compounds. In conclusion, our results demonstrate that EOs can exhibit severe toxic properties, already at low concentrations. Therefore, a detailed toxicological assessment is highly recommended for each EO and single intended application.

## Introduction

Natural compounds (phytochemicals) have been a good source for new bioactive drugs for a long time and provide unique structural diversity (Cragg and Newman 2013; Lautie et al. 2020). Therefore, phytochemicals are widely used in human nutrition and health as well as in animal production for various purposes (Barros and Ferreira 2017; Faehnrich et al. 2016; Hashemi and Davoodi 2011; Murakami and Ohnishi 2012; Rautiainen et al. 2016; Steiner and Syed 2015; Thomford et al. 2018). However, detailed information about possible negative effects of phytochemical addition on animal and human health is often lacking. Within this regard, the toxic potential of phytochemicals might be underestimated, as natural compounds are not necessarily safer than other products. It is, therefore, of critical importance to assess the toxicological properties of such substances.

The EU legislation provides that principles of Replacement, Reduction and Refinement (3Rs) should be considered systematically when animals are used for scientific purposes in the EU. With the Directive 2010/63, the European Union Reference Laboratory for alternatives to animal testing (EURL ECVAM) at the European Commission’s Joint Research Centre (JRC) was formally established with a mandate to support the development, validation and international acceptance of alternative methods. Furthermore, the Regulation on cosmetic products (1223/2009), REACH (2007/2006) and Classification, Labelling and Packaging (CLP) (1272/2008) are examples of EU legislation that require the replacement of animal testing. However, there is no mandatory use of alternative methods in the field of novel food and feed additives, which have to undergo a scientific-based safety assessment. Such supplements, before being placed on the market, are currently evaluated by scientific panels, which deliver opinions on the safety and toxicity, mainly based on the available literature data (von Holst et al. 2016). Hence, there is a great general demand for toxicity testing, and within this regard, alternative in vitro and in vivo approaches to traditional animal testing are gaining momentum, in order to reduce or even replace animal tests with validated alternatives where possible (Taylor 2018). Among them, cell and tissue culture models, the hen’s egg test on the chorioallantoic membrane (HET-CAM) as well as the *Caenorhabditis elegans* (*C. elegans*) model have gained considerable attention as alternative screening platforms to evaluate the toxicity of bioactive compounds in various context (Haselgrubler et al. 2017; Hunt 2017; Piersma 2004; Scheel et al. 2011).

Cell and tissue culture-based toxicological assays are essential tools in safety evaluation and their introduction and validation has greatly reduced the number of animals used for toxicity assessment (Araújo et al. 2014). Despite several advantages of cell-based in vitro assays, they possess major limitations. Most important, cell culture systems can only provide first insights into in vivo conditions, as they never completely model the complex physiology present in an intact organism. Thus, the use of in vitro tests alone for toxicological assessment might lead to over- or underestimation of the toxicological properties resulting in unjustified restrictions and safety attestation of compounds, respectively (Oleaga et al. 2016; Pamies and Hartung 2017). Unlike toxicity testing using cell cultures, alternative in vivo models can provide data from a whole organism, mimicking the complexity of interacting metabolism, homeostasis and signalling mechanisms that are present in mammals.

Within this context, *C. elegans* toxicity assays might represent an intermediate between in vitro and mammalian testing (Hunt 2017). It has been frequently shown that *C. elegans* models for determining acute (LC_50_) and developmental toxicity are as predictive as rat or mouse models (Boyd et al. 2010, 2016; Harlow et al. 2016; Hunt et al. 2018, 2012). In combination with a range of advantageous traits (e.g., short life and reproduction cycle, robust, easy and cheap to maintain large populations), *C. elegans* has been widely used in various research fields such as developmental biology, aging, neurobiology and most recently in toxicology (Haag et al. 2018; Honnen 2017; Hunt 2017; Leung et al. 2008; Litke et al. 2018; Nance and Frokjaer-Jensen 2019).

Besides knowledge about acute and developmental toxicity, information on mucous membrane irritation potential represents an important constituent of hazard identification of chemicals and products, as this information is used for risk assessment and management (e.g., for occupational and consumer safety) (Scheel et al. 2011). For this purpose, the HET-CAM has gained acceptance as an alternative to the rabbit eye irritation test (Draize test) for the assessment of the mucous membrane irritation potential (Barile 2010), and has been extensively used to evaluate the irritation potential of different substances and products (Derouiche and Abdennour 2017; Marquardt et al. 2010; McKenzie et al. 2015; Rajpal Deshmukh et al. 2012; Scheel et al. 2011; Steiling et al. 1999).

Essential oils (EOs) are aromatic, mainly volatile compounds extracted from different plant parts, such as leaves, flowers, roots, barks, seeds, etc. (Aziz et al. 2018). Currently, about 3000 different EOs are known, mainly consisting of a complex mixture of different volatile and non-volatile compounds such as terpenes, phenolics, alcohols, acids, esters, epoxides, aldehydes, ketones, amines and sulfides (Stevanovic et al. 2018). EOs have been used for a long time because of their anti-bacterial, -viral, -fungal, and -parasitical properties in pharmaceutical, cosmetic, food and feed industries (Aziz et al. 2018; Bakkali et al. 2008; Franz et al. 2010). Interestingly, many EOs can be found on the U.S. Food and Drug Administration’s (FDA) Generally Recognized as Safe (GRAS) list (21CFR182.20), which permits the use of EOs for different products such as cosmetics, food and feed. However, recent studies generated contradictory findings of the toxicity of EOs in vitro and in vivo, also demonstrating that some EOs already show toxic properties at very low concentrations. Effects such as respiratory disorders, mucous membrane irritation, acute toxicity, reproductive toxicity and organ toxicity were discussed within this regard (Bakkali et al. 2008; Horky et al. 2019; Mehdizadeh and Moghaddam 2018; Sandner et al. 2020a). Thus, the toxic potential must be first investigated when EOs are intended to be used for therapeutic aims, or being incorporated in cosmetic, food and feed products. Due to the high variability of the active substance content, viscosity, and hydrophobic properties, toxicity testing of EOs still remains challenging. Furthermore, well characterized and standardized protocols for reliable molecular and toxicological investigations using alternatives to animal testing are still lacking.

Multiple toxicity endpoint analysis is required for an accurate prediction of the adverse effects of compounds on living systems. Here, we report on a robust strategy for the assessment and prediction of the toxicological properties of EOs using different alternative in vitro and in vivo approaches. Rosemary, citrus and eucalyptus essential oils were selected for this study, as they were commonly used in a variety of different products, such as in cosmetics, and as food and feed supplements (de Oliveira et al. 2019; Dhakad et al. 2018; Dosoky and Setzer 2018; Hesabi Nameghi et al. 2019; Mathlouthi et al. 2012; Ozogul et al. 2015; Raskovic et al. 2014; Reyer et al. 2017; Tyagi et al. 2014).

## Materials and methods

### Reagents

EOs under study were obtained from Delacon Biotechnik GmbH (Engerwitzdorf, Austria). All chemicals necessary for cultivation of *C. elegans* were purchased from AppliChem GmbH (Darmstadt, Germany). Bacterial food source (OP50) was obtained from LabTIE International (Leiden, Netherlands).

### Cell culture

HeLa and Caco-2 cells were purchased from ATCC (Manassas, USA). STF1 cells were a kind gift from Sebastian Springer (Jacobs University Bremen, Germany). Caco-2 and STF1 cells were maintained in Dulbecco’s modified Eagle’s medium (DMEM), Hela cells were kept in RPMI medium. All media were supplemented with 100 µg/mL penicillin, 100 µg/mL streptomycin, and 10% FBS (all Biochrom GmbH, Berlin, Germany). Cells were grown at 37 °C in a humidified atmosphere (≥ 95%) with 5% CO_2_.

### Cell culture-based cytotoxicity assay

The cytotoxic effects of EOs used in this study were evaluated by using a resazurin-based in vitro toxicology assay (Sigma-Aldrich, Schnelldorf, Germany), according to the manufacturer’s instructions. Briefly, cells (Hela: 40,000, Caco-2: 120,000, STF1: 40,000 cells/well) were seeded into 96-well plates, grown to 90% confluency, and incubated with EOs at different concentrations (0.0016–1% [v/v]) for 24 h at 37 °C. Subsequently, the cells were washed and incubated with medium containing 10% resazurin for 2 h. The concentrations of the reduced form of resazurin (resorufin) were then determined using a microplate reader in fluorescence mode (544 nm excitation and 590 nm emission; POLARstar Omega, BMG LABTECH, Ortenberg, Germany). Data were analyzed using the Omega MARS Data analysis software package (BMG LABTECH, Ortenberg, Germany). Cell viability was normalized to untreated cells grown under the same conditions. Each test substance was measured in triplicate.

### *C. elegans* strains and maintenance

*C. elegans* strains were cultivated as described (Stiernagle 2006) and maintained at 20 °C. All strains (wild-type Bristol N2, DC19 [*bus-5(br19*)], CF1553 [*sod-3::GFP*], VP596 [*gst-4::GFP*], BC14926 [*cyp-14a3::GFP*], TJ356 [*daf-16::GFP*] and LG326 [*skn-1::GFP*] were obtained from the *C. elegans* Genetics Center (CGC, University of Minnesota, USA).

### Preparation of NGM agar plates for essential oil treatment experiments

Uniform and stable dispersion of the oils was ensured by applying an agar dilution method. Therefore, a defined volume of the EOs was mixed with molten NGM agar in tubes. Subsequently, the tubes were thoroughly vortexed and kept at 55 °C in a thermoshaker (Thermal Shake lite, VWR) until dispersion into a multiwell plate (e.g., 12-well or 24-well plate). For calculation of the EO exposure concentration, the volume of agar in each well was taken into account. For instance, mixing of 10 µL of EO with 990 µL NGM agar resulted in a final concentration of 1% EO [v/v] during exposure of nematodes.

### *C. elegans* age synchronization

To synchronize nematode populations, strains were washed from plates with M9 buffer, and residual bacteria were removed by washing worms once with M9 buffer prior to bleaching gravid adults using 1% (v/v) alkaline hypochlorite solution (1 mL 5% sodium hypochlorite, 0.5 mL 5 N NaOH, 3.5 mL dH_2_O) to obtain eggs. Wild type N2 nematodes dissolved after 11–13 min at room temperature, while *bus-5* mutant strain required 8–10 min to dissolve under constant shaking. Hypochlorite was removed by washing the eggs two times with M9 buffer. Eggs were transferred onto a new OP50 seeded NGM plate and were left to hatch overnight at 20 °C to give rise to a population of synchronized L1 larvae or were further incubated to the desired development stage.

### *C. elegans* lethality assay

Age-synchronized L4 nematodes were transferred in individual wells (~ 10 nematodes/well) of an OP50 seeded 24-well plate containing the respective concentration of EOs. After 24 h at 20 °C, the number of live and dead nematodes was counted through visual inspection using a dissecting microscope (Olympus SZX16). Worms were scored as dead when physical stimuli (e.g., touching using a small metal wire) failed to generate any response. Experiments were repeated at least on two different days and LC_50_ values were calculated from obtained dose–response curves.

### *C. elegans* reproduction assay

Age-synchronized L4 nematodes were separated in individual wells (1 nematode/well) of an OP50 seeded 24-well plate containing the respective concentration of EOs. After 72 h at 20 °C, the offspring of each nematode was counted. Experiments were repeated two times with three animals per concentration and experiment day.

### *C. elegans* development and reproductive toxicity (DART) assay

The DART assay was carried out as previously reported (Xiong et al. 2017) with minor modifications. In short, 24-well plates were prepared with NGM agar containing the respective amount of EO and were subsequently seeded with OP50. Age-synchronized L1 nematodes were then transferred onto each well (5 nematodes/well). Plates were continuously incubated at 20 °C and each individual well was imaged daily using an Olympus SZX16 microscope and a Hamamatsu Orca R2 camera until the bacterial lawn was completely consumed by the worms. The delay in bacterial lawn consumption was used for scoring the impact on development and reproduction.

### Gene expression analysis

Quantitative PCR (qPCR): Either N2 wild-type nematodes or *bus-5* mutants were synchronized and approximately 500 eggs were transferred onto OP50-seeded 12-well plates, containing the respective amount of EO. Nematodes were incubated at 20 °C for approximately 72 h until adulthood. Total RNA was then isolated via enzymatic lysis followed by RNeasy^®^ Plus Kit (Qiagen, Hilden, Germany) according to manufacturer’s instructions.

Subsequently, gene expression analysis was carried out as recently reported (Sandner et al. 2020b). In short, the mRNA expression levels of the genes involved in oxidative stress, such as glutathione peroxidase (*gpx-6*), glutathione S-transferase (*gst-4*), superoxide dismutase (*sod-3*; *sod-4*), glutamate-cysteine ligase (*gcs-1*), cytochrome P450 family (*cyp-37a1*; *cyp-14a3*), forkhead-type transcription factor (*daf-16*), protein skinhead-1 (*skn-1*), RRM domain-containing protein (*tiar-1*) and the genes encoding for heat shock proteins (*hsp-3*; *hsp-4*; *hsp-6*) were measured quantitatively by real-time PCR (C1000 Thermal Cycler and CFX96 Real-Time System, Bio-Rad Laboratories, Vienna, Austria).

A total amount of 50 ng of RNA was transcribed into cDNA using the iScript cDNA Synthesis Kit and qPCR with the iQ SYBR Green Supermix was carried out according to the manufacturer´s instructions (both from Bio-Rad Laboratories, Vienna, Austria). DNA denaturation and polymerase activation were performed for 3 min at 95 °C and followed by 40 PCR cycles. One amplification cycle was divided into three parts: denaturation at 95 °C for 10 s, annealing at 57.5 °C and extension at 72 °C for 20 s, and a plate read after each cycle. Finally, melt curve analysis was performed by gradually increasing the temperature to 95 °C to exclude the formation of primer dimers. Agarose gel electrophoresis was carried out to exclude unspecific products. The gene expression of the target genes in each experiment was normalized to the expression of multiple reference genes, namely, beta Actin (*act-1*), DNA-directed RNA polymerase II subunit RPB1 (*ama-1*) and Peroxisomal Membrane Protein-related protein (*pmp-3*). All experiments were performed on three individual days and measured in triplicates. The mean is based on a minimum of seven individual samples. In all qPCR analyses, the detected cT values were used to calculate the relative mRNA expression levels via the 2^−∆∆cT^ method (Livak and Schmittgen 2001). The oligonucleotide sequences of the primers (Microsynth AG, Balgach, Switzerland) used are shown in Table 1.

**Table 1:** Analysed genes in qPCR experiments and the oligonucleotide sequences of primers used

Genes	Forward Primer Sequence (5′–3′)	Reverse Primer Sequence (5′–3′)	Accession No
*act-1*	TGTTCCCATCCATTGTC	GCTCATTGTAGAAGGTGTG	NM_073418
*ama-1*	CTCCGTCGTTGACTGTAT	ATACCCATTCCTCGTCTTC	NM_068122
*pmp-3*	ATACGAAGCCACGGATAG	CTGTGTCAATGTCGTGAAG	NM_001269679
*gpx-6*	GAGGTAAATGGTCAGAACAC	TAACCGGCTGATCTCTTC	NM_001028197
*gst-4*	GTGCCTTACGAGGATTATAG	GTGATAGACATTGACTGACC	NM_069447
*daf-16*	GAATGGATGGTCCAGAATG	GATTCCTTCCTGGCTTTG	NM_001026423
*hsp-3*	ACTGTCGGAGGAGTTATG	GAACTTTCCGAGCTGATG	NM_076618
*hsp-4*	GGAAGATGCTGACATGAAG	CGATTACTCCTGCTTGAAC	NM_001306541
*hsp-6*	GAACCGGAAAGGAACAAC	GCAAACTCGGTCATCTTG	NM_071890
*sod-3*	GTGGTGGACACATCAATC	GCAATATCCCAACCATCC	NM_078363
*sod-4*	GGAGATACTGGAAATGGTTG	CACTTAATGAGGCAAGAGAG	NM_001268074.2
*gcs-1*	GATTCCCAGGTCTCATTTC	GCAGGATGAGATTGTACG	NM_063526.6
*cyp-37a1*	ATGGTCCTCTGGCTTTAC	GATCAGGGCATTGCTTTC	NM_064538.4
*cyp14a3*	CTCAAGGTGACGCATTTATC	GATCGCATAACTTGCTCTTC	NM_077804.3
*tiar-1*	CTACAAGAAAGCCAGGAGA	CGGACTTCGGTAATTCGT	NM_182180
*skn-1*	GCAAGAGATGCGTGATTC	GTAGGCGTAGTTGGATGT	NM_171345.4

Analysis of transgenic C. elegans strains*:* The effects on gene expression were monitored utilizing GFP transgenic *C. elegans* strains containing *sod-3*, *gst-4, cyp-14a3, skn-1* and *daf-16* genes. Synchronized worms (*skn-1* and *daf-16* nematodes were seeded as unsynchronized population to reduce stress upon bleaching procedure) were grown on OP50 seeded NGM 12-well plates containing the respective concentration of the EO. Worms were incubated at 20 °C for 3 or 72 h before fluorescence gene expression analysis. Single worms were transferred onto 3% agarose pads and anesthetized using a 1 mM levamisole solution for approximately 5 min to avoid nematode motion. GFP fluorescence was imaged by a CCD camera (Orca-R2, Hamamatsu, Japan) using a 2 × air objective on an Olympus SZX16 stereomicroscope equipped with a LED illumination system (CoolLED pE-300^white^) and GFP filter set.

### HET-CAM model

Fertilized hen’s eggs (Lohmann classic brown chicken) were obtained on day zero from a local breeder and were further incubated at 38 °C and 60% relative humidity for 10 days as previously reported (Haselgrubler et al. 2018a; Haselgrubler et al. 2018b). The eggs were automatically and constantly turned, checked for fertilization via candling, and the area of the air bladder was marked. At the day of the experiment, the eggshell above the chorioallantoic membrane (CAM) was removed. Subsequently, the inner membrane directly in contact with the CAM was moistened with 2 mL of 0.9% saline solution. The inner membrane was then carefully removed using forceps, without causing injury to the CAM, followed by the application of 500 µL of the test substance (EOs, or 0.1 N NaOH and saline solution as controls) directly onto the CAM. Any lysis, haemorrhaging and coagulation over a time period of 5 min was documented and compared to control groups. Images were recorded using a stereomicroscope (Olympus SZX16). After the short time exposure, the eggs were placed into a sealed bag and shock-frozen for subsequent incineration. Each test was repeated at least four times. Evaluation of the test results was carried out at fixed time intervals of 0.5, 2 and 5 min, as previously reported (Derouiche and Abdennour 2017).

Experiments performed with non-hatched avian embryos in the first two-thirds of embryonic development time are not considered an animal experiment according to the Directive 2010/63/EU.

### Gas chromatography

Identification and relative quantitation of main constituents of EOs under study was carried out using a Thermo Trace 1300 GC coupled to a Thermo ISQ 7000 MS (Thermo Fisher Scientific, Waltham, MA, US) (Pitsch 2020). Prior analysis, EOs were diluted 1:100 with methyl-tert-butyl ether. Chromatographic separation of EOs was achieved using a TRACE TR-5MS column (0.25 mm, 0.25 µm, 30 m; Thermo Fisher Scientific, Waltham, MA, US). Injector port temperature was kept at 200 °C. GC column temperature was kept at 45 °C for 1 min and was increased from 45 °C to 210 °C at a rate of 5 °C∙min^−1^ and held for 5 min. During measurements, transfer line was kept at 220 °C and ion source at 200 °C, respectively. GC was operated with helium (99.999%) at a constant flow rate of 1.0 mL/min. Each sample was determined in triplicate via 1:20 split injection of 1.0 µL. Total ion current (TIC) mode from m/z 50–500 was used for measurement. Data processing was carried out with Chromeleon 7.2.10 software (Thermo Fisher Scientific, MA, US). Identification of single constituents was carried out using standard substances and by comparing mass spectra obtained from the total ion chromatogram with NIST and MoNa mass spectrometry data library.

### Estimation of LD_50_ values

Estimation of log LD_50_ in mg/kg for substances with unknown molecular weight was carried out as previously described (ICCVAM 2006a, b; Stokes et al. 2008) using the following log regression formula:

$${\text{log}}\,{\text{LD}}_{{{50}}} \left( {{{{\text{mg}}} \mathord{\left/ {\vphantom {{{\text{mg}}} {{\text{kg}}}}} \right. \kern-\nulldelimiterspace} {{\text{kg}}}}} \right){ = 0}{\text{.372*log}}\left( {{{{\mu g}} \mathord{\left/ {\vphantom {{{\mu g}} {{\text{ml}}}}} \right. \kern-\nulldelimiterspace} {{\text{ml}}}}} \right){ + 2}{\text{.024}}$$

### Statistics

Statistical analysis war performed using GraphPad Prism (GraphPad Software, San Diego, CA, US; version 8.0.2). Two-sided *t* tests were applied to compare two experimental groups. ANOVA followed by Dunnett’s multiple comparison test was used to compare more than two groups. Significant *p* values were indicated as * (≤ 0.05), ** (≤ 0.01), *** (≤ 0.001) or **** (≤ 0.0001). Figures were prepared using CorelDraw 2019 (Corel Corporation, Ottawa, Ontario, Canada).

## Results

### Human in vitro cell culture experiments

Cytotoxicity is associated with acute health effects and depicts therefore a key factor in many prevalent toxicological modes-of-action. It covers many general mechanisms of toxicity common to most cell types, for example, disruption of cell membrane structure or function, inhibition of mitochondrial function, disturbance of protein turnover, and disruption of metabolism and energy production (Andrew 2014; Prieto et al. 2019). For a first round toxicity screen of EOs, we used a resazurin (Alamar Blue)-based in vitro toxicology approach. Alamar Blue has been widely used to assess cell viability and cytotoxicity in a range of biological and environmental systems (Rampersad 2012).

**Fig. 1:**
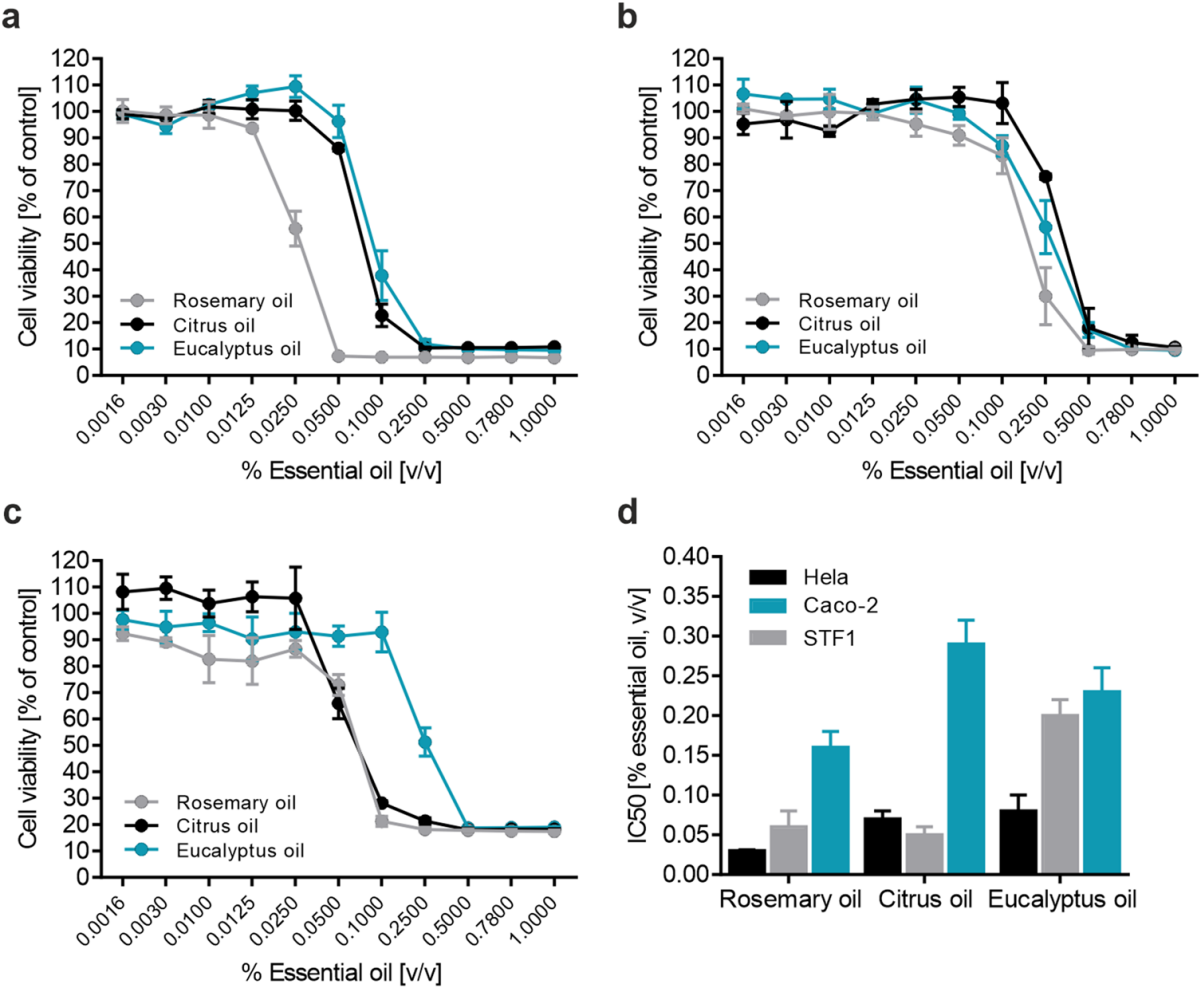
In vitro cytotoxicity of different EOs. Various human cell lines were seeded into 96-well plates (Hela: 40.000, Caco-2: 120.000, STF1: 40.000 cells/well) and grown over night at 37 °C. Cells were subsequently treated with the indicated EOs and varying concentrations for 24 h. Cell viability was measured using a resazurin-based in vitro toxicology assay. Cell viability of **a** Hela cells, **b** Caco-2 cells, and **c** STF1 cells. **d** Calculated IC_50_ values. Error bars are based on the SE of 3 independent measurements

The effects of EOs on cell viability were investigated in three different human cell lines (Hela, Caco-2 and STF1 cells) over a large concentration range, as indicated in Fig. 1. Prominent variations in cell viability upon EO treatment could be detected in a concentration-dependent manner for the three cell lines (Fig. 1a-c), with Hela cells being the most sensitive cell model. Caco-2 cells turned out to be the most robust cell line under investigation. Furthermore, significant differences in the calculated IC_50_ values were obtained (Fig. 1d), not only depending on the cell line, but also on the EO. IC_50_ values ranged tenfold from 0.03 ± 0.001% [v/v] (rosemary oil treated Hela cells) to 0.29 ± 0.03% [v/v] (citrus oil treated Caco-2 cells) of applied EO. With a mean IC_50_ value (average of all three cell lines) of 0.08 ± 0.06% [v/v], rosemary oil exhibited the highest toxicological properties based on basal cytotoxicity assessment, followed by citrus (0.13 ± 0.11% [v/v]) and eucalyptus oil (0.17 ± 0.06% [v/v]).

As simple 2D cell culture models can only provide a simplified view on the complex physiological mechanisms of higher order organisms, the toxicological properties of EOs were further investigated using alternative in vivo approaches.

### Alternative in vivo experiments

#### Plate-based *C. elegans* toxicity assay

For in-depth investigation of the toxicological effects of EOs in vivo, the N2 wild-type *C. elegans* as well as an optimized *bus-5* mutant strain was used. The *bus-5* mutant was recently described to combine features of enhanced compound permeability and chemical sensitivity with negligible fitness consequences (Xiong et al. 2017) and, therefore, represents a convenient strain especially for sensitized toxicity assessment. Due to the hydrophobic properties and volatile EO constituents, experiments were carried out in a multi-well plate format on solid media, as commonly used liquid substrates would have led to phase separation of the EO/water emulsion system, especially during long-term treatments.

**Fig. 2:**
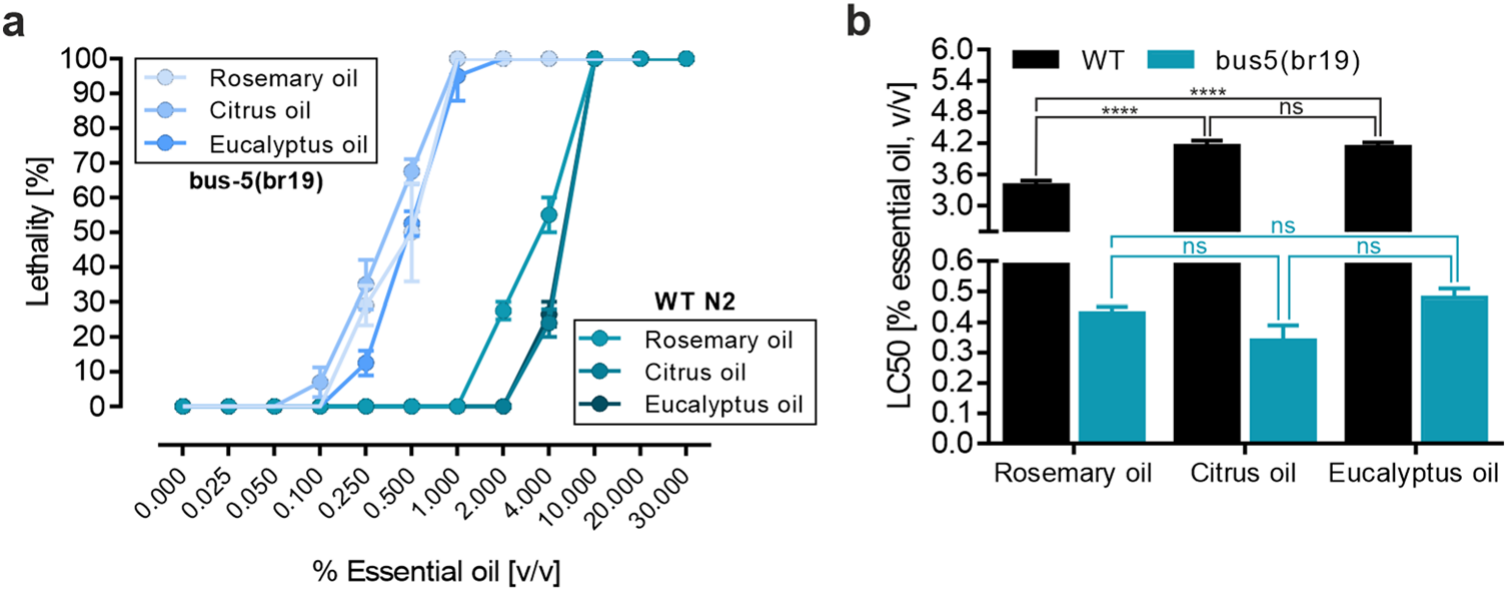
Comparison of lethality of wild type C. *elegans* and *bus-5* to different EOs. **a** Age-synchronized L4 nematodes were treated for 24 h with indicated concentrations of EOs and the number of live and dead nematodes was counted through visual inspection. **b** Comparison of calculated LC_50_ values. Error bars are based on the SE of six independent experiments measured on two different days. **** *p* < 0.0001 for comparison of LC_50_ values within indicated groups. *ns* not significant

To verify the obtained in vitro cytotoxic effects under more physiological conditions, EOs were further characterized using a *C. elegans* lethality assay. Hence, the wild-type N2 as well as the *bus-5* mutant strain were treated with various concentrations of tested EOs for 24 h and the respective LC_50_ values were calculated. The results of the lethality assay are shown in Fig. 2. Similar to the in vitro toxicology assay, the lethality was concentration dependent, for both, the N2 wild-type strain as well as for the *bus-5* mutant (Fig. 2a). The potency for lethality, as represented by LC_50_ values, was significantly higher in the N2 wild-type strain for the rosemary oil, when compared to citrus and eucalyptus oil. No significant differences were obtained for the *bus-5* mutant strain (Fig. 2b). Noteworthy, mean LC_50_ values appeared to be tenfold higher for wild-type *C. elegans*, which again confirms the previously described enhanced chemical sensitivity of the *bus-5* strain. Furthermore, LC_50_ values obtained with the sensitized *bus-5* strain were in good accordance with the IC_50_ values generated with the more robust Caco-2 cell model.

**Fig. 3:**
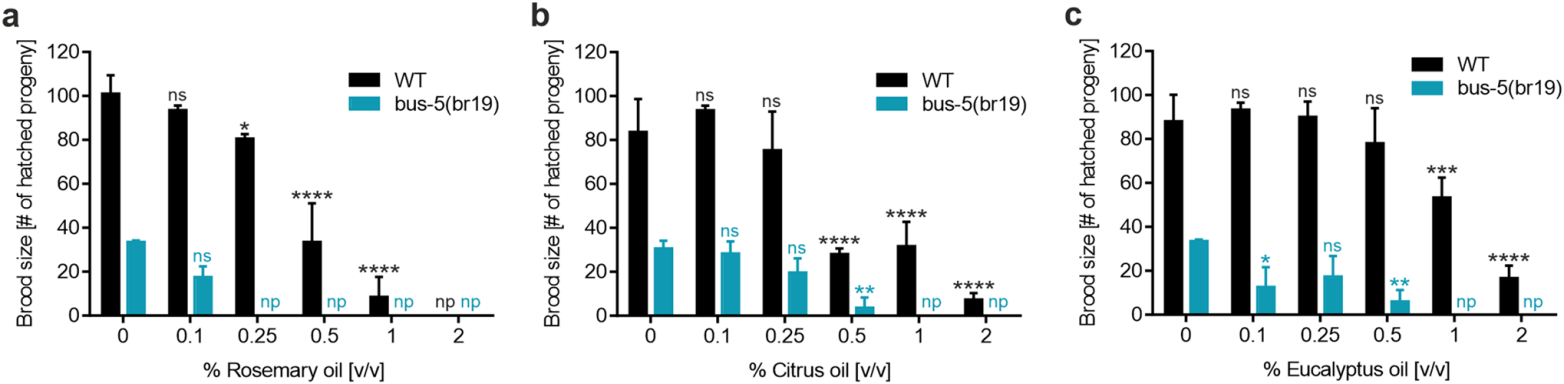
Effects on brood size of wild type *C. elegans* and *bus-5* following 72 h exposure to **a** rosemary oil, **b** citrus oil and **c** eucalyptus oil at indicated concentrations. Error bars are based on the SE of six experiments measured on two different days. * *p* < 0.05, ** *p* < 0.01, *** *p* < 0.001 and **** *p* < 0.0001 for comparison of brood size upon EO treatment within indicated groups. *ns* not significant; *np*, no progeny

Besides acute toxicity, as assessed by the lethality assay, the effect on organism reproduction represents an important parameter in chemical hazard analysis. Nematode reproduction was scored as a function of the number of offspring (brood size) after 72 h of EO treatment (Fig. 3). Again, rosemary oil showed the most prominent toxic effects, with a significant reduction in the number of N2 wild-type offspring at a concentration of 0.25% [v/v] of EO, when compared to the control group (Fig. 3a). *bus-5* mutant offspring was already slightly reduced at 0.1% [v/v] of EO, whereas no progeny was detected for higher concentrations of rosemary oil. For citrus and eucalyptus oil, significantly reduced brood size was obtained at 0.5–1% [v/v] of EO for N2 wild-type nematodes (Fig. 3b, c). At concentrations above 1% [v/v] of EO, no *bus-5* mutant offspring could be detected for citrus and eucalyptus oil.

**Fig. 4:**
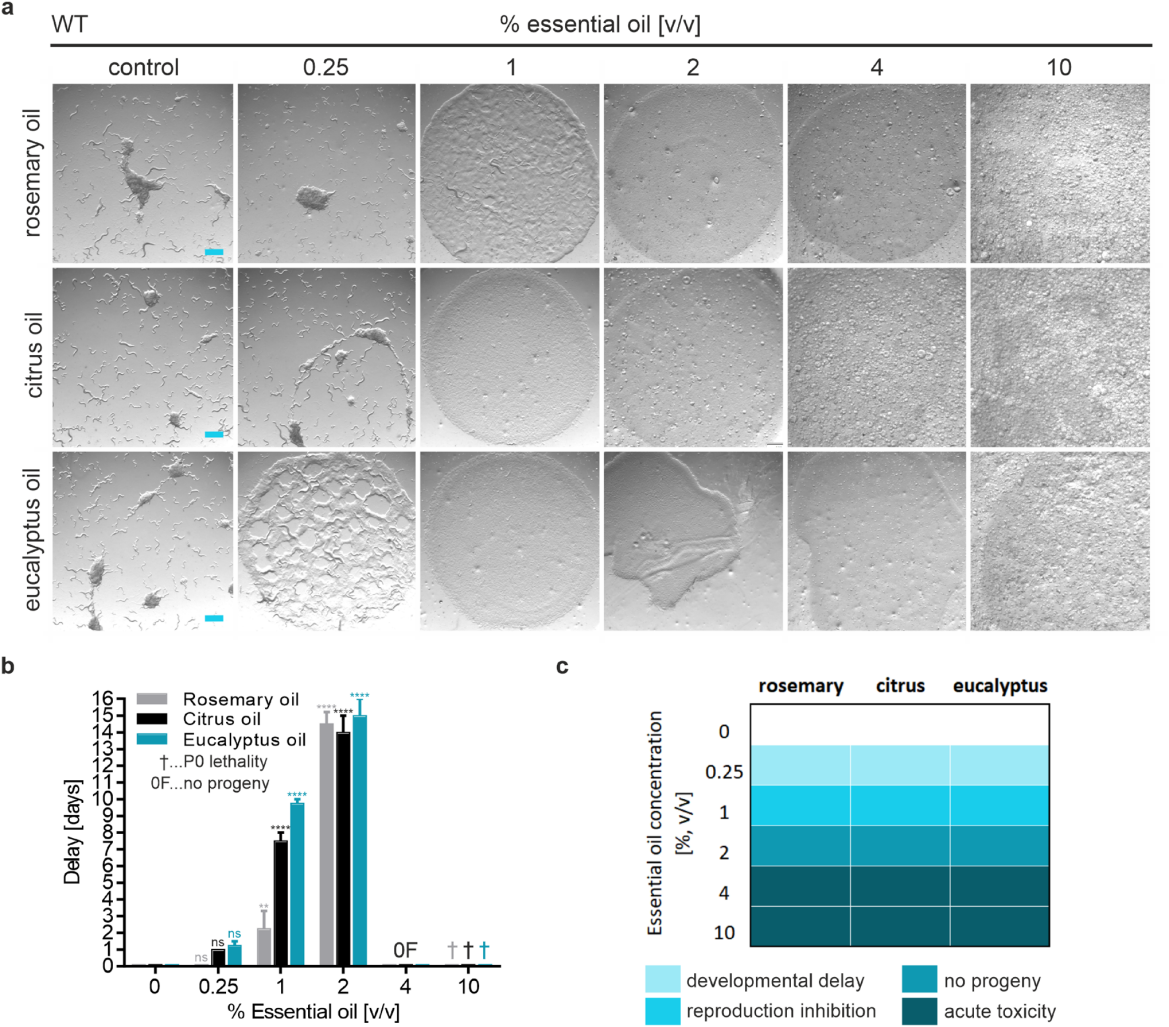
Quantitative assessment of DART in the presence of different concentrations of indicated EOs using wild type *C. elegans*. **a** Newly hatched nematodes (5 individual L1 animals) were plated onto NGM agar (containing the respective amount of essential oil), seeded with bacterial food in a 24-well plate format and allowed to grow and develop. Wells were imaged on day 6–7, when control animals plated in the absence of EOs had depleted the bacterial food source. Scale bar = 500 µm. **b** For quantitation of the delay in bacterial food source consumption, wells were scored twice a day for food depletion and the day that control worms depleted the food source was defined as day 0. Error bars are based on the SE of three independent experiments. ** *p* < 0.01, and **** *p* < 0.0001 for comparison of delay in bacterial lawn consumption upon EO treatment. *ns* not significant. **c** Comparative heatmap for different toxicity parameters as observed in daily DART scoring

**Fig. 5:**
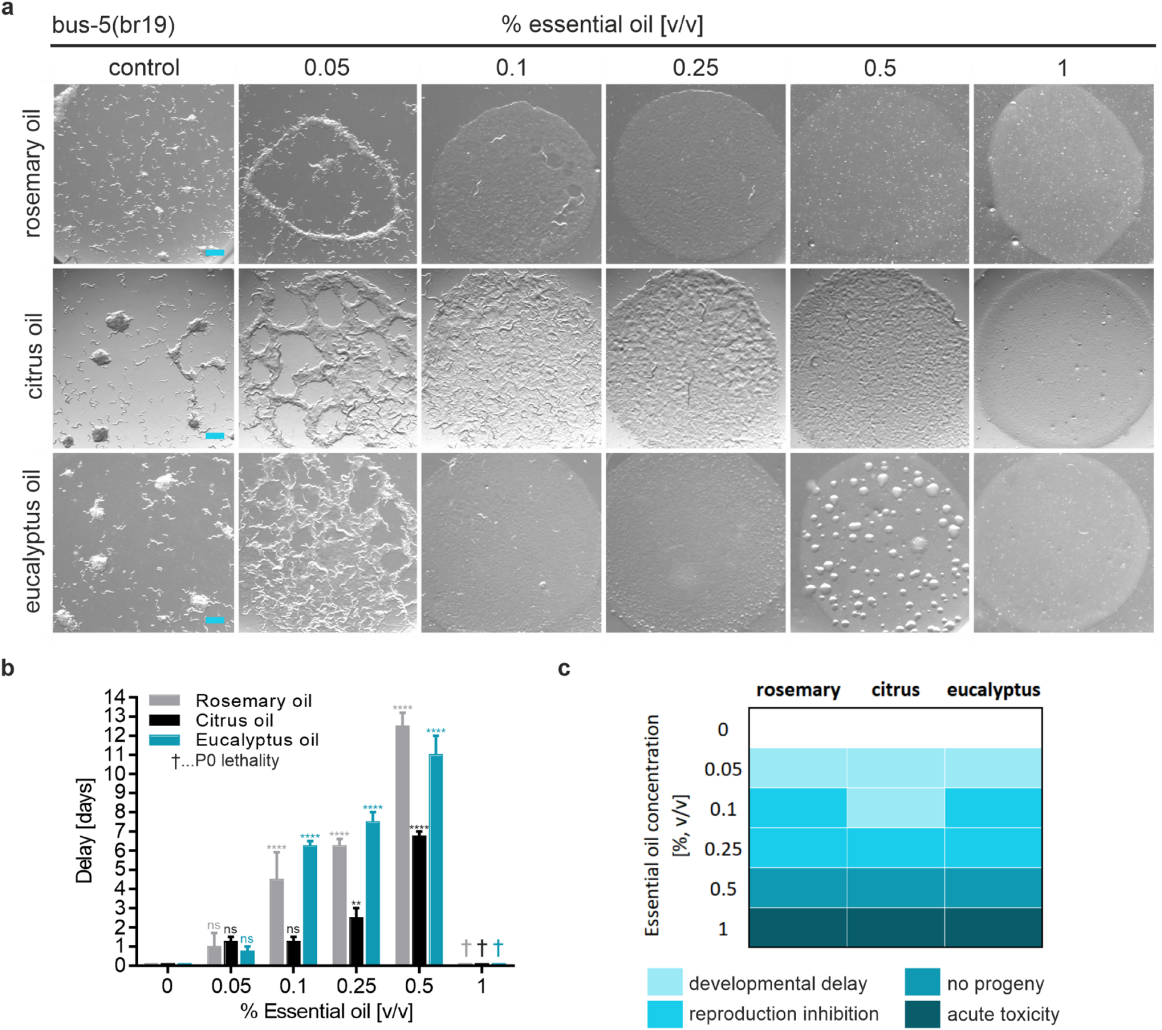
Quantitative assessment of DART in the presence of different concentrations of indicated EOs using the sensitized strain *bus-5*. **a** Newly hatched nematodes (5 individual L1 animals) were plated onto NGM agar (containing the respective amount of essential oil) seeded with bacterial food in a 24-well plate format and allowed to grow and develop. Wells were imaged on day 6–7, when control animals plated in the absence of EOs had depleted the bacterial food source. Scale bar = 500 µm. **b** For quantitation of the delay in bacterial food source consumption, wells were scored twice a day for food depletion and the day that control worms depleted the food source was defined as day 0. Error bars are based on the SE of three independent experiments. ** *p* < 0.01, and **** *p* < 0.0001 for comparison of delay in bacterial lawn consumption upon EO treatment. *ns* not significant. **c** Comparative heatmap for different toxicity parameters as observed in daily DART scoring

For direct evaluation of the effects of chronic EO exposure on *C. elegans* fitness, an adapted plate-based assay was used for scoring of acute toxicity and the impact on development and reproduction (Figs. 4, 5) (Xiong et al. 2017). As an objective numerical readout parameter of *C. elegans* size and health, the delay in the time span of bacterial lawn consumption was scored for EO treated and untreated nematodes. Thus, the day at which the strain grown in the absence of EOs completely depleted the bacterial lawn was set to zero, and delays induced by EO exposure were noted. Figure 4a shows representative images of the developmental delay of N2 wild-type nematodes treated with different concentrations of EOs under study. Similar scoring results were obtained for all three EOs (Fig. 4b, c). Some delay in food depletion was apparent at 0.25% [v/v] of EO indicating an inhibitory effect on *C. elegans* development, whereas a significant inhibition of nematode reproduction could be detected above 1% [v/v]. Acute toxicity (no progeny and no consumption of bacterial lawn) was observed at EO concentrations above 4% [v/v]. The experimental procedure was also carried out with *bus-5* nematodes, as shown in Fig. 5. Here, a developmental delay was already detected at a fivefold lower concentration of 0.05% [v/v] of EO, when compared to N2 wild-type worms. *bus-5* nematode reproduction inhibition occurred at 0.1% [v/v], and acute toxicity was scored above 1% [v/v] (Fig. 5b, c).

**Fig. 6:**
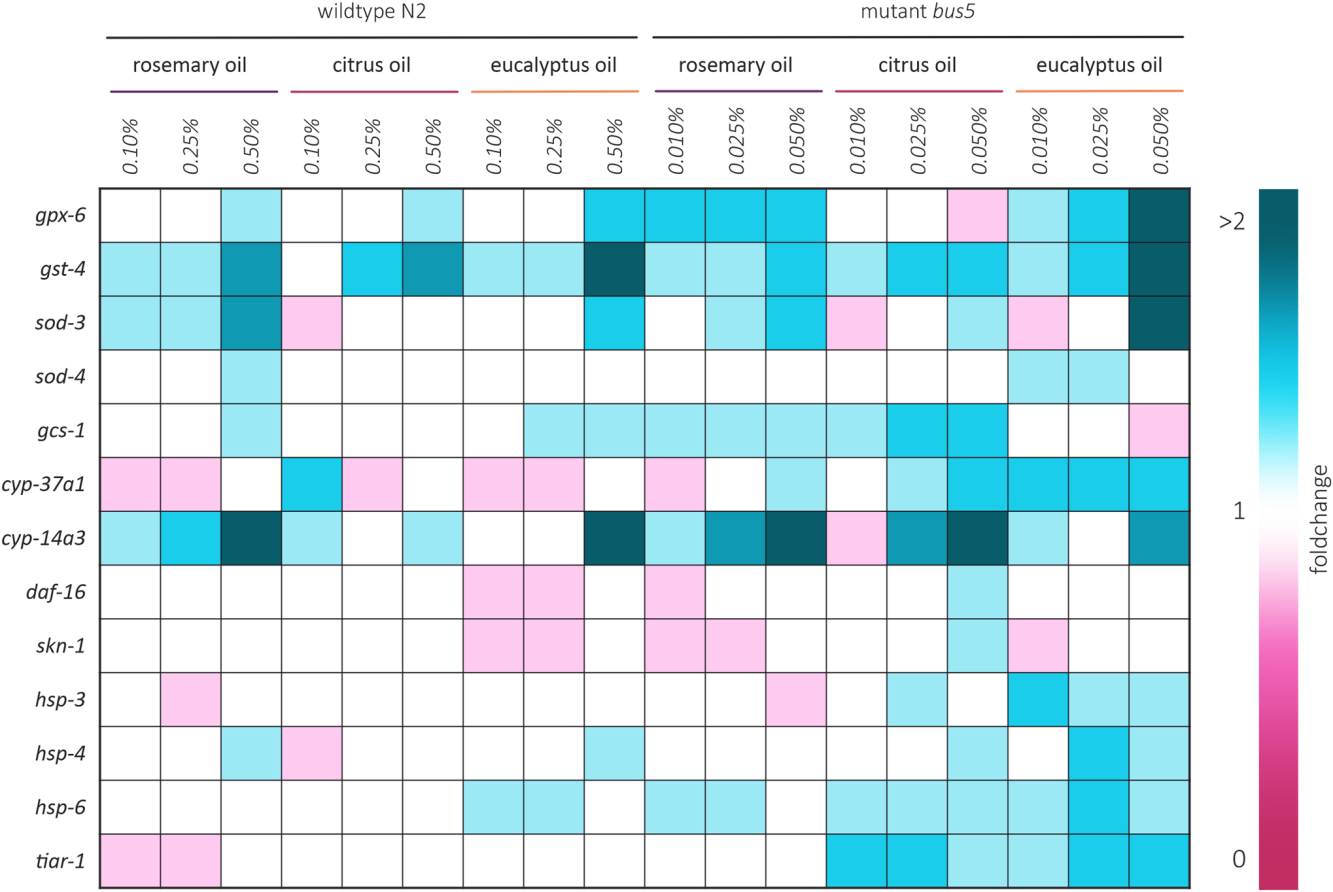
mRNA expression pattern of evaluated genes in wild type *C. elegans* and *bus-5*. Changes in gene expression upon EO treatment are shown as relative fold-change in relation to control experiments

The results described above indicated that EOs exhibited toxic properties at already low concentrations. To unravel the molecular mechanisms responsible for the observed effects at the transcriptional level, we measured the relative expression levels of 13 different key stress response genes by qPCR in wild-type *C. elegans* and *bus-5* nematodes (Fig. 6). EO concentrations for gene expression analysis experiments were chosen based on preceding results in nematode fitness tests. Therefore, wild-type *C. elegans* was treated with 0.1–0.5% of EO, and *bus-5* nematodes with tenfold lower concentrations. The selected concentrations were shown not to be acute toxic, but significantly influenced parameters such as development and reproduction (compare Figs. 2, 3, 4, 5). Generally, the impact of EO treatment on mRNA expression exhibited a comparable trend in wild-type and *bus-5* nematodes, respectively. The most significant changes in the relative expression levels were detected for the xenobiotic stress gene *cyp-14a3*, which showed an increased upregulation in relative gene expression for the highest EO concentrations ranging from 1.3- to 3.6-fold (mean 2.1 ± 0.8), when compared to the control group. Consistently, a further xenobiotic stress gene, the *gst-4* glutathione S-transferase, was found to be upregulated 1.3- to 2.2-fold (mean 1.8 ± 0.3). Furthermore, oxidative stress genes such as *gpx-6* and *sod-3* were influenced by the EO treatment, exhibiting significantly upregulated mRNA levels, particularly for the highest EO concentrations. Interestingly, the relative expression levels of two main transcription factors, *daf-16* and *skn-1*, remained unaffected or even a slight downregulation was detected. Similar results were obtained for heat-shock genes (*hsp-3*, *hsp-4*, *hsp-6*). Figure 6 summarizes the effects for all EOs and 13 stress-response genes in form of a descriptive heatmap. Taken together, gene expression analysis clearly indicated that EOs significantly alter expression patterns of important stress-response genes, already at low concentrations.

**Fig. 7:**
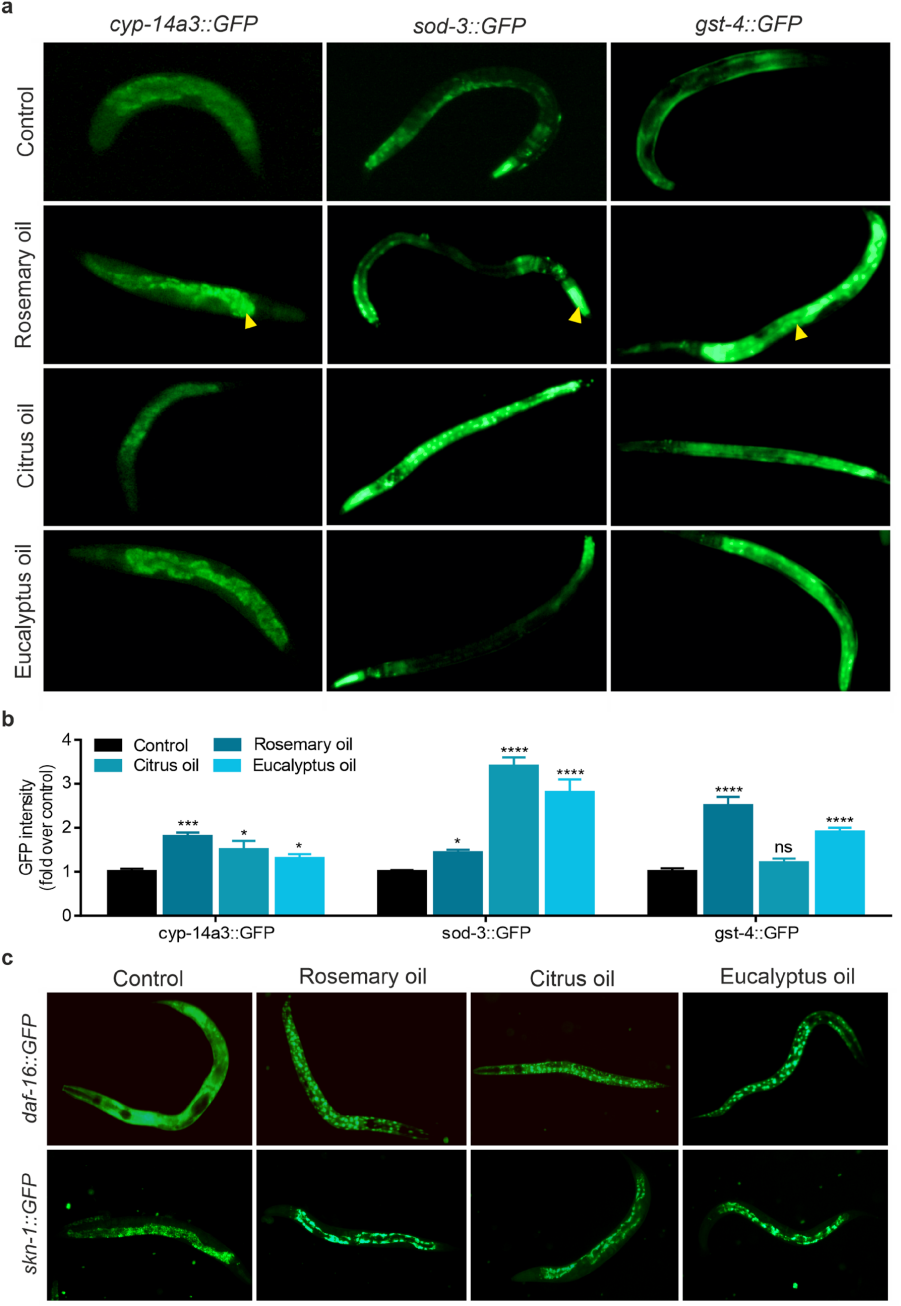
Expression and localization analysis of transgenic genes using GFP-reporter strains. Synchronized nematodes were treated with 0.25% [v/v] of EO or left untreated (control) for 72 h prior imaging. **a** Representative fluorescence microscopy images of *cyp-14a3::GFP*, *sod-3::GFP* and *gst-4::GFP* transgenic nematodes are shown. Arrow heads indicate characteristic expression pattern of the respective reporter gene. **b** Quantitation of indicated GFP-reporter gene intensity relative to control. Error bars are based on the SE of two independent experiments with at least 20 worms in each treatment. * *p* < 0.05, *** *p* < 0.001 and **** *p* < 0.0001 for comparison of EO treatment with control groups; ns, not significant. **c** Representative fluorescence microscopy images showing subcellular translocation behaviour of *daf-16::GFP* and *skn-1::GFP* upon three hours of EO (0.25% [v/v]) treatment

qPCR results were further confirmed by examination of transgenic *C. elegans* strains carrying GFP-reporter genes for the most responsive targets as identified by mRNA expression analysis (*cyp-14a3::GFP*, *gst-4::GFP* and *sod-3::GFP*). Figure 7a depicts representative images of adult transgenic nematodes treated for 72 h with 0.25% [v/v] of indicated EO. All three genes were found to be significantly upregulated after exposure to EOs, again indicating a stress response at already low EO concentrations (Fig. 7b). In addition to the aforementioned transgenic strains, we further investigated the impact of EOs on *daf-16* and *skn-1* genes by use of respective GFP-reporter nematodes (*daf-16::GFP* and *skn-1::GFP*). As *daf-16* and *skn-1* expression levels were found to be unaltered all over the different EO treatments, we aimed for the investigation of putative differences in subcellular expression patterns, as *daf-16* and *skn-1* were previously reported to undergo nuclear translocation upon oxidative stress triggering (Henderson and Johnson 2001; Kahn et al. 2008). As shown in Fig. 7c, nuclear translocation was visible for both, *daf-16::GFP* and *skn-1::GFP* positive nematodes, already after three hours of 0.25% [v/v] of EO treatment. On the contrary, the GFP signal was found to be homogenously distributed in the cytosol in untreated nematodes.

#### HET-CAM

**Fig. 8:**
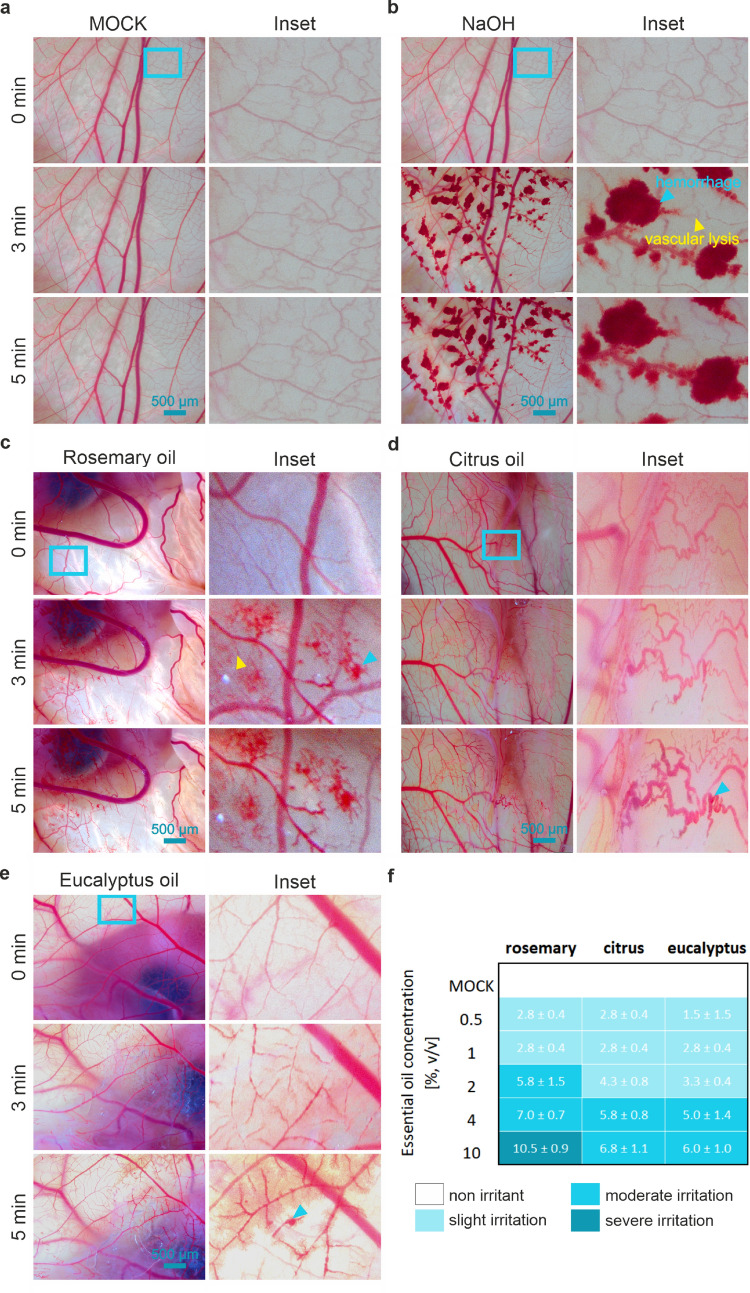
Principle of HET-CAM scoring assay and evaluation of EO irritation potential. Representative CAM photographs illustrating the effect on the membrane over a time period of 5 min of 0.5 ml saline solution (0.9%, negative control) (**a**), 0.5 ml NaOH solution (0.1 M, positive control) (**b**), and 10% EO treatment (**c**–**e**). Blue and yellow arrow heads indicate haemorrhage and vascular lysis, respectively (as shown in b). **f** Scoring of acute mucous membrane irritation potential of EOs

Data on mucous membrane irritation are generally required for the hazard identification of chemicals. Therefore, the HET-CAM was used as an alternative method to the Draize rabbit eye test for EO irritation testing. First, as a basis of the evaluation procedure, control experiments were performed (Fig. 8a, b). For this purpose, healthy membranes were treated with 0.9% saline solution (negative control) or with 0.1 M NaOH (positive control), respectively. Application of the saline solution produced no visual response over the 5-min observation period (irritation score [IS] = 0) (Fig. 8a). On the contrary, 0.1 M NaOH resulted in severe, instant haemorrhage, which further increased over the time period of 5 min. Additionally, vascular lysis was detected, grading this solution as a severe irritant (IS = 15) (Fig. 8b). Similar to the control experiments, the short-term effect of EOs on mucous membrane irritation was investigated (Fig. 8c-f). Therefore, membranes were treated with the different EOs with varying concentrations (0–10% [v/v]) and irritation scoring was carried out based on the CAM appearance over a 5-min period. Generally, the EO irritation potential increased in a concentration-dependent manner. Rosemary oil was identified as the most potent irritant, exhibiting a slight irritation potential between 0.5% and 1% [v/v], moderate irritation between 2 and 4% [v/v], and severe irritation at 10% [v/v]. A comparable pattern was detected for citrus and eucalyptus oil with a slightly reduced irritation potential when compared to the rosemary oil. Figure 8c-e shows representative photographs of the CAM at 0, 3, and 5 min after 10% EO addition.

#### Chemical characterization of EOs

**Fig. 9:**
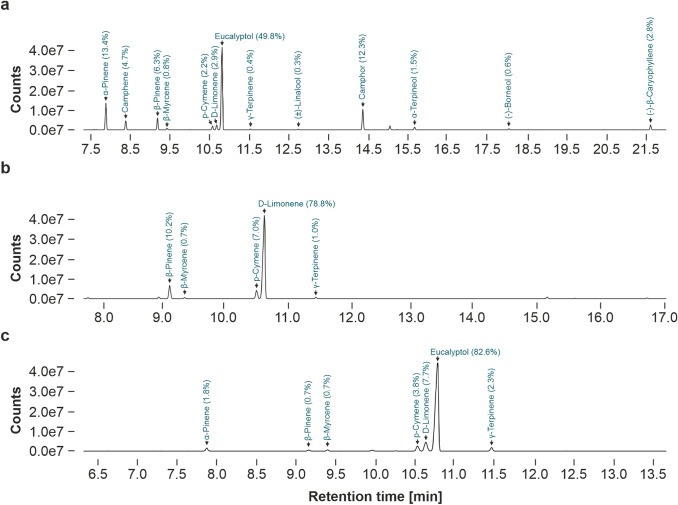
GC–MS chromatograms of rosemary (**a**), citrus (**b**), and eucalyptus essential oil (**c**). Numbers in brackets represent the relative amount of single essential oil constituents

To identify the main compounds of EOs under study, GC–MS analysis was carried out (Fig. 9). For rosemary oil, 13 different components were identified, representing ~ 98% of the total oil (Fig. 9a). The major constituents identified were eucalyptol (49.8%), α-pinene (13.4%), camphor (12.3%) and β-pinene (6.3%). In citrus oil, five different compounds were identified, representing a 97.7% of the total oil (Fig. 9b), with d-limonene (78.8%), β-pinene (10.2%) and p-cymene (7.0%) as the main constituents. Seven different compounds were found in eucalyptus oil (Fig. 9c), whereas eucalyptol (82.6%), d-limonene (7.7%), p-cymene (3.8%) and γ-terpinene (2.3%) made 96.4% of the total oil.

#### Prediction of LD_50_ values

Most toxicity studies available carried out with alternative approaches to animal tests aimed in the identification of adverse toxic reactions on a molecular level or were conducted as toxicity ranking screens with high throughput. However, to reduce unnecessary animal testing, a correlation between in vitro IC_50_/LC_50_ and in vivo LD_50_ values is indispensable. We, therefore, predicted oral LD_50_ values based on the calculated IC_50_ (cell culture) and LC_50_ (*C. elegans*) values using a validated IC_50_-LD_50_ log regression approach for estimating starting doses for acute oral systemic toxicity testing (ICCVAM 2006a, b). Table 2 depicts a summary of all calculated mean IC_50_ and LC_50_ values as well as the corresponding predicted LD_50_ values. Furthermore, a comparison with already known LD_50_ values for EOs under study is shown. Importantly, we could detect a high correlation of the predicted mean LD_50_ values with data available obtained from oral toxicity screens in rats for EOs under study, indicating that this prediction method might have the potential to completely replace animal testing in some cases.

**Table 2:** Estimation of oral LD_50_ based on measured IC_50_/LC_50_ values obtained with alternative in vitro and in vivo models

		Rosemary oil			Citrus oil			Eucalyptus oil		
			Mean LD_50_ predicted [g/kg]	LD_50_ literature^1^ [g/kg]		Mean LD_50_ predicted [g/kg]	LD_50_ literature [g/kg]		Mean LD_50_ predicted [g/kg]	LD_50_ literature [g/kg]
Cell culture	Mean IC_50_ [mg/mL]	0.72	**2.8**	**2.0–5.5**	1.2	**2.9**	**2.8–5.1**	1.5	**3.1**	**2.5–4.4**
	Predicted LD_50_ [g/kg]	**1.2**			**1.5**			**1.6**		
WT nematode	Mean LC_50_ [mg/mL]	30.6			37.8			36.9		
	Predicted LD_50_ [g/kg]	**4.9**			**5.2**			**5.2**		
*bus5* nematode	Mean LC_50_ [mg/mL]	3.6			3.1			4.3		
	Predicted LD_50_ [g/kg]	**2.2**			**2.1**			**2.4**		

^1^Rat LD_50_ (oral) values (for all three oils) for comparison were obtained from material safety data sheets of commercially available EOs

## Discussion

EOs possess manifold bioactive properties and are therefore continuously being tested for various applications such as natural pesticides (Pavela and Benelli 2016), food preservatives (Pandey et al. 2016), alternatives to antibiotics in animal feed (Stevanovic et al. 2018), compounds in aromatherapy (Reis and Jones 2017) and cosmetic ingredients (Sarkic and Stappen 2018). However, due to large variations in chemical composition, mainly depending on ambient growth conditions, genetic diversity, and extraction procedures, commercial exploitation and exploration of EOs is difficult (Horky et al. 2019; Tammar et al. 2019). This might also be the reason for contradictory findings of EO toxicity in vitro and in vivo. For a comprehensive risk assessment, dose–response evaluation, different time of exposure, identification of the mechanisms of toxicity and main EO constituents should be considered. Within this context, alternatives to animal testing, such as different in vitro cytotoxicity and in vivo models are gaining momentum (Pamies and Hartung 2017; Taylor 2018).

In a first phase of toxicity evaluation, EOs under study were tested using a robust in vitro cell culture cytotoxicity assay. Depending on the cell line, culture model (2D vs. 3D) and EO variety, large deviations in cytotoxic effects of EOs were reported (Horky et al. 2019). Similar results were obtained in our study, with an approximately tenfold difference in obtained IC_50_ values, ranging from 0.03 to 0.29% [v/v], also depending on cell type as well as on EO. As simple cell culture models lack of important features such as organ functionality, appropriate tissue architecture, physiological environment and are especially prone to artefacts (e.g., over- or underestimation of toxic effects), they should not be used as ‘stand-alone’ approaches, as many questions in toxicology remain very complex and depend on network responses of the biological model. To simulate organ- and tissue-like culture conditions, novel cell culture technologies including stem-cell derived human cells, microfluidics, 3D cultures and organ-on-chip approaches emerged within the last years (Pamies and Hartung 2017). However, such methods are rarely available in laboratories, demand for specialized equipment, do not allow for higher throughput investigations and are challenging in implementation and validation. In this context, the nematode *C. elegans* represents an attractive alternative testing model for predictive toxicology, as it allows for toxic exposure information in a whole animal with many genetic, developmental, neuronal and toxic mode of action processes that are conserved with mammals (Hunt et al. 2020). Furthermore, *C. elegans* can be handled with standard in vitro equipment and techniques, also allowing for high-throughput investigations.

Whereas several studies are available describing and reporting on toxic effects of EOs using different human and animal derived cell lines, as well as animal models (Horky et al. 2019), toxicological assessment using alternative approaches to animal tests, such as *C. elegans*, is lacking. We, therefore, provide extensive investigation of the effects of EOs on multiple endpoints using a plate-based toxicology approach. As *C. elegans* cuticle was recently reported to present a barrier to chemical uptake and might, therefore, lead to underestimation of toxic effects, we also included the cuticle integrity mutant *bus-5*, which showed a robust toxicity outcome and enhanced chemical sensitivity at much lower concentrations than wild-type *C. elegans* (Xiong et al. 2017). In our study, toxic effects in *bus-5* mutants were detected at approximately five to tenfold lower concentrations than in wild-type nematodes, facilitating the detection of adverse outcome that would be missed using only wild-type worms. Calculated mean LC_50_ value (0.42% [v/v]) for all EOs in *bus-5* nematodes is in accordance with the mean IC_50_ value (0.23% [v/v]) obtained for the more robust Caco-2 cell line. These results clearly indicate that the selection of appropriate model systems is of critical importance to avoid under- as well as overestimation of toxicological compound properties, which might potentially be the case for more sensitive cell lines such as Hela cells or insensitive wild-type *C. elegans*, respectively.

In the nucleus, the *daf-16* transcription factor regulates various genes associated with stress resistance or general lifespan. Previously, it was shown that phytochemicals are able to affect the nuclear translocation of DAF-16 (Abbas and Wink 2010; Duangjan et al. 2019; Yen et al. 2011). Additionally, the nuclear translocation activates genes including antioxidant enzymes (*gpx-6*, *gst-4*, *sod-3*) and catalases. Transcriptional activation of *gst-4* is also utilized as indicator of SKN-1 activity (Kahn et al. 2008). Additionally, *gcs-1* is regulated under oxidative stress which also involves accumulation of SKN-1 (An et al. 2005). In our study, we identified *gst-4*, *sod-3* and *cyp-14a3* relative gene expression to be prominently upregulated upon EO treatment (both wild-type and *bus-5*). Fluorescent *C. elegans* strains further confirmed the upregulation of the selected genes. The expression patterns (*sod-3::GFP:* pharyngeal area, *gst-4::GFP*: whole body, *cyp-14a3::GFP*: intestinal area) are in accordance with the literature (Akhoon et al. 2016; Shanmugam et al. 2017; Stefanello et al. 2015). Nuclear translocation patterns of *daf-16::GFP* and *skn-1::GFP* revealed those main pathways to be involved in regulatory mechanisms affected by the exposure of EOs. In general, the effect of nuclear translocation decreased with prolonged EO treatment (not shown) in our experiments, possibly indicating nematode adaption to chronic stress conditions. Furthermore, we cannot exclude other key response genes of other pathways (e.g.,: c-Jun N-terminal kinase (JNK) or insulin/insulin-like growth factor signalling (IIS)), which might also be involved. In addition, for *skn-1::GFP* translocation*,* it is very important to exclude autofluorescence signals (e.g. from lipofuscin), since the expression level and consequently the fluorescence intensity in this transgenic strain was generally very low. Hence, nuclear translocation might be masked by intestinal and other granules (Hu et al. 2017; Wang et al. 2016). Therefore, further research would be necessary to receive additional insight in the regulatory mechanisms. Additionally, subject to the intended application, other endpoints of concern, which are not covered in this study (e.g., genotoxicity, allergenicity, etc.) might deserve attention in safety evaluations of EOs.

EOs are known as mucous membrane, eye, and skin toxicants. The most important adverse reactions that may occur include irritation, sensitization and photosensitization (Ali et al. 2015). Within this regard, the HET-CAM test method showed potential to refine and reduce animals used in irritation testing (Barile 2010). Previous studies using the HET-CAM as a model test system revealed irritating effects of different EOs (Demirci et al. 2005; Moura do Carmo et al. 2012; Reichling et al. 2006). Based on these findings, we aimed in the investigation of the mucous membrane irritating potential of the three EOs under study. A minor irritation potential was already detected at 0.5% [v/v] of applied EO, which was further raised with increasing EO concentration (e.g., moderate irritation potential for all three EOs at 4% [v/v]). Interestingly, the concentration of 0.5% [v/v] is similar to the LC_50_ value obtained in *bus-5* nematodes (0.4% [v/v]), whereas the level of moderate mucous membrane irritation at 4% [v/v] correlates with the mean LC_50_ value of WT nematodes (3.9% [v/v]). Thus, the HET-CAM model might also represent a complementary tool for the prediction of LD_50_ in vivo.

Besides developmental and reproductive toxicity, information on acute toxicity is used to describe a substance’s putative harmful effect. Within this regard, the oral LD_50_ value is still frequently used as a standard parameter, mostly obtained from animal tests. Importantly, the extrapolated LD_50_ is often unreliable when used in human health risk assessment, as a wide variability between different species is reported (Pereira and Tettamanti 2011). Nevertheless, the LD_50_ represents an important parameter to describe the toxic potential of a substance of interest. As the LD_50_ is usually expressed as the mass of substance administered per unit mass of the test subject, typically as mg/kg of body mass, we aimed in the prediction of the EOs LD_50_ based on our alternative in vitro and in vivo approaches. We therefore used the log regression equation, which was originally developed to predict starting doses for the acute toxicity testing in animals based on *in vitro* cytotoxicity studies (Stokes et al. 2008). Interestingly, the predicted mean LD_50_ values for EOs under study were in good agreement with available LD_50_ values of commercial EO extracts, indicating that this approach might not only be suited to predict starting doses for toxicity testing in animals, but also to directly estimate the LD_50_. However, detailed investigations with various chemical substances as well as alternative in vitro and in vivo approaches in combination with large-scale regression analysis will be necessary to elaborate such a validated and reliable prediction model.

As the EO composition can be subjected to large variations and to facilitate the comparison of the obtained toxicity results with data reported in the literature and with upcoming studies, the chemical composition of EOs was characterized by GC–MS. Quantitation of analytes was carried out by peak area normalization and data were expressed in terms of the percentage of the total peak area. Identified single constituents represented > 95% of the total peak area, with eucalyptol (rosemary [48.8%] and eucalyptus oil [82.6%]) and d-limonene (citrus oil [78.8%]) being the most abundant monoterpenes present. In general, the main identified components of EOs under study are consistent with previous reports (Ben Hsouna et al. 2017; Sienkiewicz et al. 2013; Tyagi et al. 2014). For EOs available on the market, detailed data on chemical composition is frequently lacking. This missing information impedes toxicological predictions and, therefore, chemical characterization is highly recommended for each single used EO and intended product.

Animal testing still represents the ‘gold-standard’ in toxicity and safety assessment of industrial chemicals, pharmaceuticals, cosmetics, and agrochemicals. However, as shown in this study, holistic alternative approaches with sophisticated toxicity analysis exhibit the potential to reduce or even completely replace animal testing.

## Data Availability

Not applicable.
